# Genetic association studies in critically ill patients: protocol for a systematic review

**DOI:** 10.1186/s13643-023-02401-3

**Published:** 2023-12-13

**Authors:** Eline G. M. Cox, Wenbo Zhang, Peter H. J. van der Voort, Gerton Lunter, Frederik Keus, Harold Snieder

**Affiliations:** 1 Department of Critical Care, University Medical Center Groningen, Groningen, 9713 GZ the Netherlands; 2 Department of Epidemiology, University Medical Center Groningen, Groningen, 9713 GZ the Netherlands

**Keywords:** Genetics, Critically ill patients, Intensive care unit, Genome-wide association study, Candidate gene study

## Abstract

**Introduction:**

Patients in the intensive care unit (ICU) are highly heterogeneous in characteristics, their clinical course, and outcomes. Genetic variability may partly explain the variability and similarity in disease courses observed among critically ill patients and may identify clusters of subgroups. The aim of this study is to conduct a systematic review of all genetic association studies of critically ill patients with their outcomes.

**Methods and analysis:**

This systematic review will be conducted and reported according to the *HuGE Review Handbook V1.0*. We will search PubMed, Embase, and the Cochrane Library for relevant studies. All types of genetic association studies that included acutely admitted medical and surgical adult ICU patients will be considered for this review. All studies will be selected according to predefined selection criteria, evaluated and assessed for risk of bias independently by two reviewers. Risk of bias will be assessed according to the *HuGE Review Handbook V1.0* with some modifications reflecting recent insights. We will provide an overview of all included studies by reporting the characteristics of the study designs, the patients included in the studies, the genetic variables, and the outcomes evaluated.

**Ethics and dissemination:**

We will use data from peer-reviewed published articles, and hence, there is no requirement for ethics approval. The results of this systematic review will be disseminated through publication in a peer-reviewed scientific journal.

**Systematic review registration:**

PROSPERO CRD42021209744.

**Supplementary Information:**

The online version contains supplementary material available at 10.1186/s13643-023-02401-3.

## Background

Patients with (multiple) organ failure in the hospital are admitted to the intensive care unit (ICU) [1]. These critically ill patients suffer high rates of adverse outcomes resulting in death or, when they survive, often a reduced quality of life [2–4]. The worldwide burden of critical illness is high and is expected to increase with aging populations [4]. Patients admitted to the ICU may have a wide variety of primary diagnoses for which they were initially admitted to the ICU. As a result, the patients in the ICU are highly heterogeneous. In addition, their critical illness may have an unpredictable course with divergent outcomes even when the initial diagnosis is the same. They can either show a quick recovery or they deteriorate with progressive multi-organ failure. Also, patterns of critical illness and (multi)organ failure may show remarkably similar patterns in disease courses, irrespective of the primary underlying disease [5].

In 2001, a draft sequence of the Human Genome became available which resulted in an explosion of studies that identified genetic determinants of diseases [6, 7]. Single-nucleotide polymorphisms (SNPs) are the most commonly occurring type of variant in the genome and also the most frequently studied variants in genetic association studies. The development of a haplotype map based on common genetic variants (i.e., SNPs) in the HapMap project has contributed to the understanding of the patterns of diversity across the human genome [8–10]. It has been shown that genetic variability is able to identify clusters of subgroups among heterogeneous patient groups [11]. Similarly, we hypothesize that genetic variability may partly explain the variability of disease courses observed among critically ill patients. Only observational studies of genetic associations can address this hypothesis.

Genetic association studies fall into two broad categories: candidate gene studies and genome-wide association studies (GWASs) [12]. Candidate gene studies are hypothesis based; genes are selected either by their location in a region of linkage or on the basis of other evidence [12]. While such studies did propose many associations, these were usually not successfully validated in independent cohorts, likely due to small and heterogeneous study samples, random error in exploration studies [13], or insufficient power to detect associations with polymorphisms [14]. The introduction of GWASs facilitated an unbiased survey of common SNPs and hypothesis-free discovery analyses [13]. Although GWASs also have limitations, they represent an important advance for discovery of genetic variants influencing diseases [15].

The discovery of novel genetic loci using hypothesis-free GWAS approaches may help to identify their underlying biology and improve our understanding of common causal pathways in critical illness and (multi)organ failure. However, most genetic association studies and reviews have focused on the evaluation of risk factors within single specific diseases or syndromes in critical care, such as sepsis or acute kidney injury [16–18]. In addition, an overview of all available existing evidence on genetics and a wide range of diseases and/or outcomes that are common in the ICU, such as (multiple) organ failure, sepsis, septic shock, multiple lung diseases, and mortality, is lacking. Therefore, the aim of this study is to conduct a systematic review of all genetic association studies in critically ill patients.

## Methods

This systematic review referred to the *HuGE Review Handbook V1.0* (guidelines for systematic review and meta-analysis of gene disease association studies) and its updated version that included GWAS to create a quality assessment tool [19, 20]. Several new domains were included in the tool to cover GWAS-related content, bringing it more in line with current insight in genetic research. The protocol will adhere to the PRISMA-P checklist [21].

### Selection criteria

#### Types of studies

All types of genetic association studies will be included. We will include candidate gene studies and GWASs. Articles will be excluded if they do not report original data (e.g., review studies); if they are conference abstracts, case reports/series, and editorials; or if they are not conducted in humans. Association studies of (genome-wide) methylation or gene expression will also be excluded.

Only studies which included adult (≥ 18 years old) critically ill patients admitted to an ICU will be included in this review. Studies that include patients who developed multiple organ failure, heart failure, liver failure, acute kidney injury, renal insufficiency, sepsis, septic shock, acute respiratory distress syndrome, pulmonary edema, acute lung injury, and COVID-19 will all be included. Studies including patients with other diseases than mentioned above (e.g., neurological diseases such as stroke or subarachnoid hemorrhage) will be excluded as the majority of these patients will typically not be admitted to the ICU. Studies in cohorts of patients admitted to the ICU after planned surgery will also be excluded, while studies including patients after acute surgery will be considered for this review.

We will include genetic studies that evaluate the association with mortality, following the definition according to the original authors’ terminology. We will also include the genetic association study if any other patient-relevant outcome measure was reported for the primary analysis in the original study. Genetic studies that evaluate associations with surrogate outcomes or laboratory values, e.g., leucocytes, proteins, or cytokines, will be excluded for evaluation in this review.

### Identifying studies

PubMed, Embase, and the Cochrane Library will be searched for relevant studies. The search strategies are listed in Supplementary material 1. All identified publications will be imported into EndNote, and duplicates will be removed. Two reviewers will independently evaluate the titles and abstracts of all the studies generated by the search strategies for eligibility according to predefined selection criteria. At least two reviewers will assess the full-text articles of the preliminary selected publications to see if they meet all inclusion criteria and to extract data. The reference lists of the articles selected will be scrutinized for additional studies. Any disagreements will be resolved through discussion, and if consensus cannot be reached, a third reviewer will be consulted. A database will be created for screening of eligible articles. The number of hits screened, the number of full-text studies retrieved, and the number of excluded studies will be reported using the PRISMA flow chart.

### Data collection and analysis

The data of included articles will be extracted and registered in a database in Excel. We will extract the following data from each report: first author, year of publication, country of study, title, study design (candidate gene study/GWAS), type of genetic marker, genotyping method, sample size, gender, age, ethnicity, definition of the disease, and effect sizes such as odds ratios (ORs) [20]. After extraction, the data will be checked by a second reviewer to minimize the risk of transcription errors.

A quality assessment tool based on the *HuGE Review Handbook V1.0* and its updated version with GWAS content will be used for assessment of the quality of individual studies [20, 21]. The risk of bias of each study will be assessed by 12 questions representing the following domains: selection bias, information bias, confounding, multiple testing and replication, and Hardy–Weinberg equilibrium. Details of the quality assessment tool are shown in Table 1. Two reviewers will assess the risk of bias of all included papers, independently. Any disagreements will be resolved through discussion, if necessary, including other reviewers. We will consider studies that are classified as low risk of bias in all domains as being at “overall low risk of bias.” Studies with one or more of these domains assessed as “some concerns” or “high risk of bias” will be judged as overall “high risk of bias.”

**Table 1:**
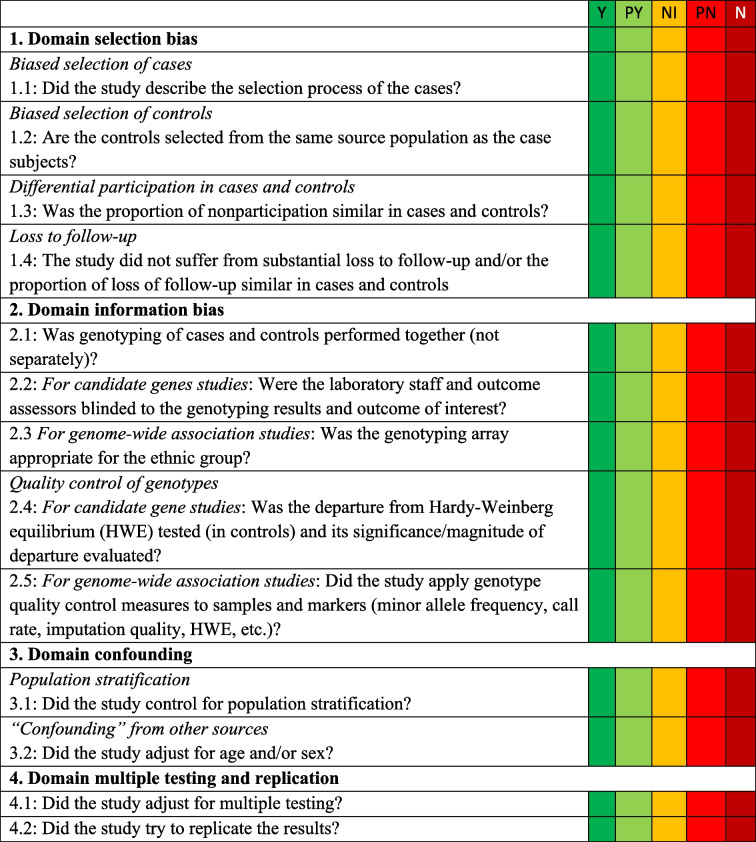
Risk-of-bias assessment for genetic association studies based on the HuGE Review Handbook V1.0*

*Abbreviations*: *Y* yes, *PY* probably yes, *NI* no information, *PN* probably no, *N* no

*Each domain has signaling questions. Only if answers to all signaling questions within a domain are “yes” or “probably yes,” then the domain will be assessed as “low risk of bias.” If the answers to any of the signaling questions within a domain are “no information,” then the domain will be assessed as “some concerns.” If one or more of the answers to signaling questions within a domain are “probably no” or “no,” then the domain will be assessed as “high risk of bias.” We will consider studies that are assessed as having “low risk of bias” in all domains as being at “overall low risk of bias.” Studies with one or more of these domains assessed as “some concerns” or “high risk of bias” will be judged as being at “overall high risk of bias”

### Data synthesis and statistical analysis

A descriptive synthesis will be carried out including the major findings of each study. We will describe the characteristics of the studies designs and the patients included in the studies. Further, we will report the genes observed to be associated with the outcomes, classified according to the study design (candidate gene study or GWAS). We will present a systematic overview of the evidence and interpret and discuss the observations of the included studies.

## Discussion

Critical illness leads to a major burden of disease for patients and relatives and generates high costs for hospitals. Such burden of critical illness is higher than generally appreciated and will continue to increase in the future [22]. Critical illness in itself is difficult to define but is characterized by ICU admission due to some degree of organ failure. ICU patients are very heterogeneous reflected by differences in ICU admittance diagnoses, differences in disease courses, and variations in outcomes. Previous genetic studies and reviews in the field of ICU medicine have focused on genetic associations within single specific diseases or syndromes.

In contrast with the wide variation in types of ICU patients, there are remarkable similar patterns of organ failure during critical illness, also across different admitting diagnoses, irrespective of the primary underlying disease. We hypothesize that there may be shared genetic variation associated with the occurrence and severity of organ failure. The present systematic review will be able to give an overview of genetic loci which are associated with some degree of organ failure and will enable easier access to published results by the public and researchers alike. Such results might encourage external validation of earlier findings. These results will help direct choice of genes of interest for future genetic studies on organ failure and/or critically ill patients.

An important potential limitation may be that this systematic review evaluates organ failure in a wide selection of heterogenous critically ill patients with varying admitting diagnoses, which contrasts with typical genetic reviews into genetic loci associated with one specific disease. Due to the large number of outcomes, the results may, therefore, end up in a large list of potential candidate genes and loci each with limited evidence and based on small samples of patients.

A newly developed quality assessment tool will be used to report the risk of bias of the included studies. This tool was based on the *HuGE Review Handbook V1.0* with some modifications reflecting recent insights related to GWAS. While meant to improve the assessment of the risk of bias of the included studies, our modified instrument has not been applied previously and may not cover all possible sources of bias.

The quantitative results observed in different studies may be difficult to compare. A formal meta-analysis will likely not be feasible due to the heterogeneity of the study populations and outcomes and the variety in the candidate genes studied. Therefore, this study will likely present a systematic narrative overview of the evidence. If feasible, we will report the results of any meta-analysis only as a hypothesis generating explorative finding.

### Supplementary Information

**Additional file 1.** Search strategy: Pubmed Search Strategy. Embase Search Strategy. Cochrane Library Search Strategy.

## Data Availability

Not applicable.

## Abbreviations

ICU: Intensive care unit
SNP: Single-nucleotide polymorphism
GWAS: Genome-Wide Association Study
OR: Odds ratio
