# Supplementary material for: Genetic association studies in critically ill patients: protocol for a systematic review
Source: Syst Rev. 2023 Dec 13;12:233. doi: 10.1186/s13643-023-02401-3 (PMC10716946; doi:10.1186/s13643-023-02401-3)
Supplement: Supplementary file 1 — Additional file 1. Search strategy: Pubmed Search Strategy. Embase Search Strategy. Cochrane Library Search Strategy. [file 13643_2023_2401_MOESM1_ESM.docx]

**Supplementary Material. Search strategy**

# Pubmed Search Strategy

("Critical Care"[MeSH Terms] OR "Intensive Care Units"[MeSH Terms] OR icu[Title/abstract] OR “intensive care”[Title/abstract] OR icus[Title/abstract] OR “critically ill*”[Title/abstract] OR “critical ill*”[Title/abstract] OR “critical care”[Title/abstract] OR “major trauma”[Title/abstract] OR “major blunt trauma”[Title/abstract])

AND

(“Genome-Wide Association Study”[MeSH Terms] OR "Genetic Association Studies"[MeSH Terms] OR “Polymorphism, Genetic”[MeSH Terms] OR Genomics[MeSH Terms] OR "Genetic Predisposition to Disease"[MeSH Terms] OR "Genetic Research"[MeSH Terms] OR Genome[MeSH Terms] OR genetics[Mesh major topic] OR genetics[Mesh Subheading] OR “GWA stud*”[Title/abstract] OR “GWAS”[Title/abstract] OR polymorphism*[Title/abstract] OR gene[Title/abstract] OR genes[Title/abstract] OR genet*[Title/abstract] OR genom*[Title/abstract] OR genotyp*[Title/abstract] OR snp[Title/abstract] OR snps[Title/abstract] OR haplotyp*[Title/abstract] OR  “genome-wide association stud*” [Title/abstract] OR “candidate gene analys*”[Title/abstract]  OR “candidate gene association stud*”[Title/abstract] OR “whole genome sequenc*”[Title/abstract] OR “whole exome sequenc*”[Title/abstract])

AND

("Multiple Organ Failure"[MeSH Terms] OR "Shock, Septic"[MeSH Terms] OR Sepsis[Mesh Terms] OR "Liver Failure"[MeSH Terms] OR "Heart Failure"[MeSH Terms] OR "Respiratory Distress Syndrome"[MeSH Terms] OR "Pulmonary Edema"[MeSH Terms] OR "Acute Lung Injury"[MeSH Terms] OR "Renal Insufficiency"[Mesh major topic] OR “multiple organ failure*”[Title/abstract] OR MODS[Title/abstract] OR “multiple organ dysfunction*”[Title/abstract] OR “multi-organ*”[Title/abstract] OR “organ failure*”[Title/abstract] OR “organ dysfunction*”[Title/abstract] OR “liver failure*”[Title/abstract] OR “heart failure*”[Title/abstract] OR “cardiac dysfunction*”[Title/abstract] OR “left ventricular dysfunction*”[Title/abstract] OR sepsis[Title/abstract] OR “septic shock”[Title/abstract] OR “renal insufficienc*”[Title/abstract] OR “acute kidney injur*”[Title/abstract] OR AKI[Title/abstract] OR “acute lung injur*”[Title/abstract] OR “pulmonary edema”[Title/abstract] OR “acute respiratory distress syndrome”[Title/abstract] OR ARDS[Title/abstract] OR “respiratory failure*”[Title/abstract] OR  "Coronavirus Infections"[MeSH Terms] OR covid*[Title/Abstract] OR corona[Title/Abstract] OR coronavirus[Title/abstract] OR sars-CoV-2[Title/Abstract])

AND

("Cohort Studies"[MeSH Terms] OR "Case-Control Studies"[MeSH Terms] OR "cohort*"[Title/Abstract] OR "case-control"[Title/Abstract] OR "case control"[Title/Abstract])

NOT

("Animals"[MeSH Terms] NOT "Humans"[MeSH Terms]) NOT ("Review" [Publication type] OR "Case reports" [Publication type] OR "Meta-analysis" [Publication type] OR review[Title] OR "case report" [Title] OR "meta-analysis" [Title] OR "meta analys*" [Title]) NOT (("Child"[MeSH Terms] OR "Adolescent"[MeSH Terms] OR "Infant"[MeSH Terms] OR neonat*[Title] OR pediatric*[Title]) NOT "Adult"[MeSH Terms])

# Embase Search Strategy

(‘intensive care’/exp OR ‘critical care’/exp OR ‘intensive care unit’/exp OR ‘intensive care’:ab,ti,kw OR ICU:ab,ti,kw OR ICUs:ab,ti,kw OR ‘critically ill*’:ab,ti,kw OR ‘critical ill*’:ab,ti,kw OR ‘critical care’:ab,ti,kw OR ‘major trauma’:ab,ti,kw OR ‘major blunt trauma’:ab,ti,kw)

AND

(‘genome-wide association study'/exp OR ‘genetic association study'/exp OR genomics/exp OR genome/exp OR ‘genetic predisposition'/exp OR ‘genetic polymorphism’/exp OR genetics/mj OR ‘candidate gene’/exp OR ‘whole genome sequencing’/exp OR ‘whole exome sequencing’/exp OR polymorphism*:ab,ti,kw OR gene:ab,ti,kw OR genes:ab,ti,kw OR genet*:ab,ti,kw OR genom*:ab,ti,kw OR genotyp*:ab,ti,kw OR snp:ab,ti,kw OR snps:ab,ti,kw OR haplotyp*:ab,ti,kw OR ‘genome-wide association stud*’:ab,ti,kw OR  ‘GWA stud*’:ab,ti,kw OR  ‘GWAS’:ab,ti,kw OR ‘candidate gene analys*’:ab,ti,kw OR ‘candidate gene association stud*’:ab,ti,kw OR ‘whole genome sequenc*’:ab,ti,kw OR ‘whole exome sequenc*’:ab,ti,kw)

AND

(‘multiple organ failure’/exp OR sepsis/exp OR ‘septic shock’/exp OR ‘liver failure’/exp OR 'kidney failure'/exp OR ‘heart failure’/exp OR ‘respiratory distress syndrome’/exp OR ‘lung edema’/exp OR ‘acute lung injury’/exp OR ‘multiple organ failure*’:ab,ti,kw OR MODS:ab,ti,kw OR ‘multiple organ dysfunction*’:ab,ti,kw OR ‘multi-organ*’:ab,ti,kw OR ‘organ failure*’:ab,ti,kw OR ‘organ dysfunction*’:ab,ti,kw OR ‘liver failure*’:ab,ti,kw OR ‘heart failure*’:ab,ti,kw OR ‘cardiac dysfunction*’:ab,ti,kw OR ‘left ventricular dysfunction*’:ab,ti,kw OR sepsis:ab,ti,kw OR ‘septic shock’:ab,ti,kw OR ‘renal insufficienc*’:ab,ti,kw OR ‘acute kidney injur*’:ab,ti,kw OR AKI:ab,ti,kw OR ‘acute lung injur*’:ab,ti,kw OR ‘pulmonary edema’:ab,ti,kw OR ‘acute respiratory distress syndrome’:ab,ti,kw OR ARDS:ab,ti,kw OR ‘respiratory failure*’:ab,ti,kw OR  ‘Coronavirus Infection’/exp OR covid*:ab,ti,kw OR corona:ab,ti,kw OR coronavirus:ab,ti,kw OR sars-CoV-2:ab,ti,kw)

AND

(‘cohort analysis’/exp OR ‘case control study’/exp OR ‘cohort*’:ab,ti,kw OR ‘case-control’:ab,ti,kw OR ‘case control’:ab,ti,kw)

NOT

(animal/exp NOT human/exp) NOT (Review:it OR ‘Case reports’:it OR ‘Case report’:it OR ‘Meta-analysis’:it OR meta-analysis:it OR ‘conference abstract’:it OR ‘review’:ti OR ‘case report’:ti OR ‘meta-analysis’:ti OR ‘meta analys*’:ti) NOT (('child'/exp OR 'adolescent'/exp OR 'infant'/exp OR neonat*:ti OR pediatric*:ti) NOT 'adult'/exp)

# Cochrane Library Search Strategy

#1 MeSH descriptor: [Critical Care] explode all trees

#2 MeSH descriptor: [Intensive Care Units] explode all trees

#3 ((intensive care unit):ti,ab,kw OR (ICU):ti,ab,kw OR (ICUs):ti,ab,kw OR (critically ill*):ti,ab,kw OR (critical ill*):ti,ab,kw OR (critical care):ti,ab,kw OR (major trauma):ti,ab,kw OR (major blunt trauma):ti,ab,kw)

#4 MeSH descriptor: [Genome-Wide Association Study] explode all trees

#5 MeSH descriptor: [Genetic Association Studies] explode all trees

#6 MeSH descriptor: [Genome] in all MeSH products

#7 MeSH descriptor: [Genetic Predisposition to Disease] explode all trees

#8 MeSH descriptor: [Polymorphism, Genetic] explode all trees

#9 MeSH descriptor: [Genetic Association Studies] explode all trees

#10 MeSH descriptor: [Whole Genome Sequencing] explode all trees

#11 MeSH descriptor: [Exome Sequencing] explode all trees

#12 ((polymorphism*):ti,ab,kw OR (gene):ti,ab,kw OR (genes):ti,ab,kw OR (genet*):ti,ab,kw OR (genom*):ti,ab,kw OR (genotyp*):ti,ab,kw OR (SNP):ti,ab,kw OR (SNPs):ti,ab,kw OR (haplotyp*):ti,ab,kw OR (genome-wide association study):ti,ab,kw OR (candidate gene analys*):ti,ab,kw OR (candidate gene association stud*):ti,ab,kw OR (whole genome sequenc*):ti,ab,kw OR (whole exome sequenc*):ti,ab,kw)

#13 MeSH descriptor: [Multiple Organ Failure] explode all trees

#14 MeSH descriptor: [Sepsis] explode all trees

#15 MeSH descriptor: [Shock, Septic] explode all trees

#16 MeSH descriptor: [Liver Failure] explode all trees

#17 MeSH descriptor: [Renal Insufficiency] explode all trees

#18 MeSH descriptor: [Heart Failure] explode all trees

#19 MeSH descriptor: [Respiratory Distress Syndrome] explode all trees

#20 MeSH descriptor: [Acute Lung Injury] explode all trees

#21 ((multiple organ failure*):ti,ab,kw OR (MODS):ti,ab,kw OR (multiple organ dysfunction*):ti,ab,kw OR (multi organ*):ti,ab,kw OR (organ failure*):ti,ab,kw OR (organ dysfunction*):ti,ab,kw OR (liver failure*):ti,ab,kw OR (heart failure*):ti,ab,kw OR (cardiac dysfunction*):ti,ab,kw OR (left ventricular dysfunction*):ti,ab,kw OR (sepsis):ti,ab,kw OR (septic shock):ti,ab,kw OR (renal insufficienc*):ti,ab,kw OR (acute kidney injur*):ti,ab,kw OR (AKI):ti,ab,kw OR (acute lung injur*):ti,ab,kw OR (pulmonary edema):ti,ab,kw OR (acute respiratory distress syndrome):ti,ab,kw OR (ARDS):ti,ab,kw OR (respiratory failure*):ti,ab,kw  OR (covid*):ti,ab,kw OR (corona):ti,ab,kw OR (coronavirus):ti,ab,kw OR (sars-CoV-2):ti,ab,kw)

#22 MeSH descriptor: [Cohort Studies] explode all trees

#23 MeSH descriptor: [Case-Control Studies] explode all trees

#24 ((cohort*):ti,ab,kw OR (case-control):ti,ab,kw OR (“case control”):ti,ab,kw)

#25 MeSH descriptor: [Animal Experimentation] explode all trees

#26 MeSH descriptor: [Human Experimentation] explode all trees

#27 ((“review”):pt OR (“case report”):pt OR (“meta analysis”):pt OR (“abstract”):pt OR (review):ti OR (case report):ti OR (meta-analysis):ti OR (meta analys*):ti)

#28 (((child):kw OR (infant):kw OR (infant*):ti OR (neonat*):ti OR (pediatric*):ti) NOT (adult):kw)

(#1 OR #2 OR #3) AND (#4 OR #5 OR #6 OR #7 OR #8 OR #9 OR #10 OR #11 OR #12) AND (#13 OR #14 OR #15 OR #16 OR #17 OR #18 OR #19 OR #20 OR #21) AND (#22 OR #23 OR #24) NOT (#25 NOT #26) NOT #27 NOT #28
